# Omics-driven advances in the understanding of regulatory landscape of peanut seed development

**DOI:** 10.3389/fpls.2024.1393438

**Published:** 2024-05-03

**Authors:** Zhihui Wang, Yong Lei, Boshou Liao

**Affiliations:** ^1^ Key Laboratory of Biology and Genetic Improvement of Oil Crops, Ministry of Agriculture and Rural Affairs, Oil Crops Research Institute of the Chinese Academy of Agricultural Sciences (CAAS), Wuhan, China; ^2^ National Key Laboratory of Crop Genetic Improvement, National Center of Crop Molecular Breeding Technology, National Center of Oil Crop Improvement (Wuhan), Huazhong Agricultural University, Wuhan, China

**Keywords:** omics, seed development, peanut, yield, quality

## Abstract

Peanuts (*Arachis hypogaea*) are an essential oilseed crop known for their unique developmental process, characterized by aerial flowering followed by subterranean fruit development. This crop is polyploid, consisting of A and B subgenomes, which complicates its genetic analysis. The advent and progression of omics technologies—encompassing genomics, transcriptomics, proteomics, epigenomics, and metabolomics—have significantly advanced our understanding of peanut biology, particularly in the context of seed development and the regulation of seed-associated traits. Following the completion of the peanut reference genome, research has utilized omics data to elucidate the quantitative trait loci (QTL) associated with seed weight, oil content, protein content, fatty acid composition, sucrose content, and seed coat color as well as the regulatory mechanisms governing seed development. This review aims to summarize the advancements in peanut seed development regulation and trait analysis based on reference genome-guided omics studies. It provides an overview of the significant progress made in understanding the molecular basis of peanut seed development, offering insights into the complex genetic and epigenetic mechanisms that influence key agronomic traits. These studies highlight the significance of omics data in profoundly elucidating the regulatory mechanisms of peanut seed development. Furthermore, they lay a foundational basis for future research on trait-related functional genes, highlighting the pivotal role of comprehensive genomic analysis in advancing our understanding of plant biology.

## Introduction

As a source of edible vegetable oil and protein, peanut (*Arachis hypogaea* L.) is an oil and economic crop of worldwide importance. The peanut are now cultivated in more than 100 countries, mainly distributed in developing countries in Asia, Africa and South America. The global peanut production has been about 54 million tons annually, with a consistent cultivation area of approximately 31 million hectares (ha) in recent years (http://faostat.fao.org). Peanuts have a high nutritional value, as they are rich in fats (35%~60%) and proteins (22%~35%), and also provide dietary fiber, minerals, vitamins and bioactive macromolecules (Zhao et al., 2020b; Chen et al., 2021; Zhou et al., 2021). The traits of peanuts cover various characteristics such as seed weight, oil content, protein content, fatty acid composition, sucrose content, seed coat color, etc. The improvement of these traits is currently a key area of focus in in peanut genetic breeding. These traits are intricately linked to the expression and regulation of genes during seed development. Consequently, the elucidation of the peanut seed development process based on omics data has become a research hotspot in recent years, shedding light on the regulatory mechanisms governing the formation and variation of essential traits in peanut seeds.

The peanut seed development process spans from flowering to subterranean fruiting, illustrating the unique geocarpic growth habit of peanuts. In this process, the flower pollinates above ground, and then the peg, carrying the fertilized ovule, elongates and burrows into the soil to form the seed. The development process of peanut seeds is highly intricate, governed by numerous genes that regulate various seed traits such as size, weight, oil content, seed coat color, fatty acid composition, and the concentration of functional substances. Therefore, researching the regulatory genes and related molecular mechanisms involved in the peanut development process is of significant importance for the genetic improvement of peanut traits.

**Figure 1:**
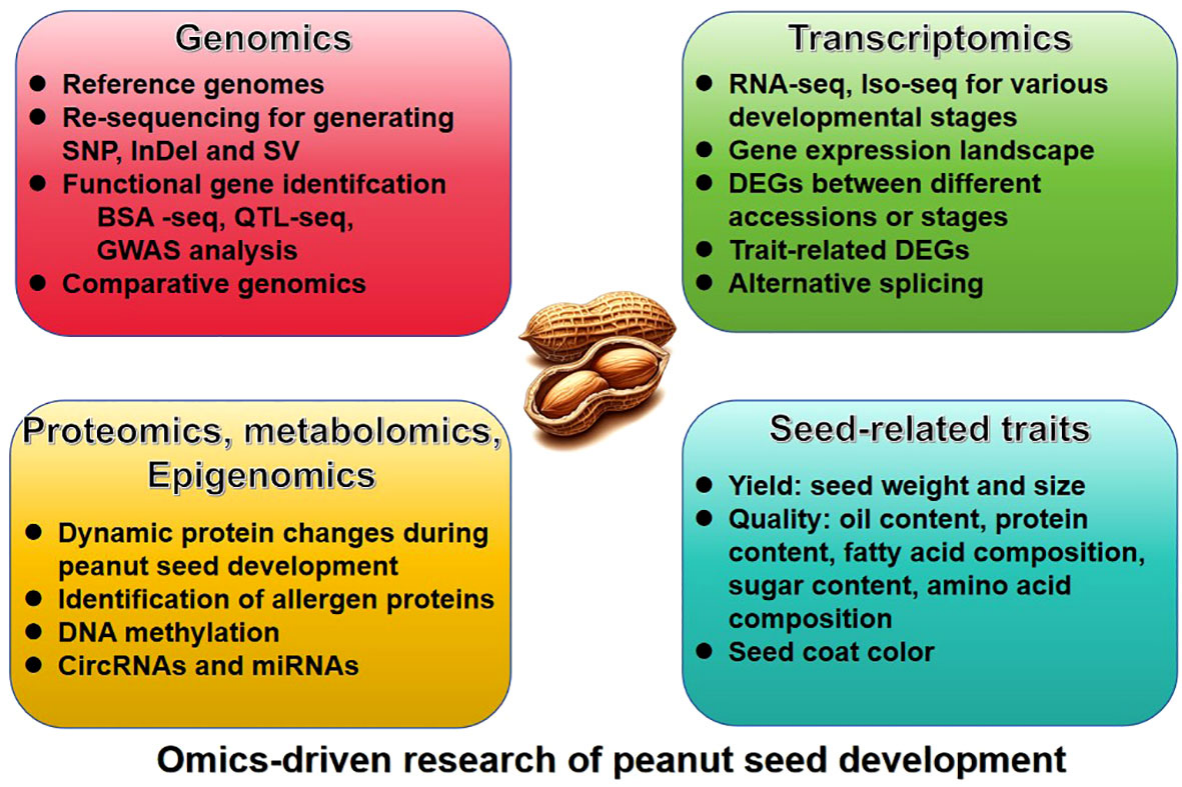
The overview of omics-driven research of peanut seed development.

The omic-technology with illumina or long sequencing reads was utilized to construct the reference genome of peanut. The genome of the diploid progenitors of cultivated peanut, *A. duranensis* and *A. ipaensis*, was sequenced first (Bertioli et al., 2016; Chen et al., 2016b; Hu et al., 2018), followed by the genome of the cultivated allotetraploid peanut *A. hypogaea* (Bertioli et al., 2019; Zhuang et al., 2019). Since the release of the peanut reference genome, significant progress have been achieved in the investigation of quantitative trait loci (QTL) mapping, expression regulation, epigenetics, and other facets pertaining to seed-related traits (**Table 1**). These advancements have been facilitated through the comprehensive analysis of diverse omics datasets, such as resequencing data, transcriptome, proteome, metabolome, and epigenome, etc (**Figure 1**).

**Table 1:** Reference list for omics-driven research on peanut seed development.

Re-sequencing data were employed for SNP genotyping to construct high-resolution genetic maps, identify quantitative trait loci (QTL), or conduct genome-wide association studies (GWAS) focusing on seed-related traits					
Omics technology	Traits/application	Reference	Omics Data	Traits	Reference
SLAF-seq	Yield-related Traits/QTL	Wang et al., 2018b	BSA-seq	Sucrose content/QTL	Guo et al., 2023
SLAF-seq	Seed weight/QTL	Zhang et al., 2019	BSA-seq	Red testa/QTL	Zhang et al., 2022b
SLAF-seq	Seed weight/QTL	Zhao et al., 2022	ddRAD-seq	Yield-related traits/GWAS	Wang et al., 2019;
SLAF-seq	Oleic and Linoleic Acid/QTL	Hu et al., 2018	ddRAD-seq	Fatty acid components/GWAS	Zhang et al., 2021
WGS-seq	Quality traits/QTL	Sun et al., 2022	Axiom_Arachis2 SNP array	Seed weight/GWAS	Zhao et al., 2022
WGS-seq	Sucrose content/QTL	Wang et al., 2024	WGS-seq	Yield-related traits/GWAS	Zhou et al., 2021
WGS-seq	Purple testa/QTL	Zhao et al., 2020b	WGS-seq	Fatty acid components/GWAS	Zhou et al., 2022
ddRAD-seq	Trans-resveratrol content/QTL	Luo et al., 2021	Axiom_Arachis2 SNP array	Fatty acid components/GWAS	Otyama et al., 2022
ddRAD-seq	Oil content/QTL	Liu et al., 2020c	SLAF-seq	Seed weight/GWAS	Zhang et al., 2017
Transcriptome, Protome, Metabolome data or multi-omics data joint analysis were employed for seed development or seed-related traits					
RNA-seq	SD	Clevenger et al., 2016	RNA-seq	Sucrose content	Li et al., 2021a
RNA-seq	SD	Gupta et al., 2016	RNA-seq	Seed coat color	Wan et al., 2016
RNA-seq	SD	Yin et al., 2013	RNA-seq	Seed coat color	Huang et al., 2020
RNA-seq	SD	Zhang et al., 2012	Proteomic	SD and lipid metabolism	Wang et al., 2016;
RNA-seq	SD	Chen et al., 2013	Proteomic	SD and allergen proteins	Li et al., 2020
RNA-seq	SD	Zhu et al., 2014	RNA-seq and DNA Methylation	Oil content	Liu et al., 2022;
RNA-seq	SD	Zhang et al., 2016	Methylation	SD and seed size	Li et al., 2023
RNA-seq	SD	Chen et al., 2016a	CircRNAs	SD and seed size	Feng et al., 2019
RNA-seq	SD	Zhao et al., 2020a	miRNA	SD	Chen et al., 2019a
RNA-seq	SD	Chen et al., 2019b	miRNA	SD	Ma et al., 2018
RNA-seq	SD	Yu et al., 2015	Metabolomics	Seed coat color	Zhang et al., 2022a
RNA-seq	SD	Liu et al., 2020a	Metabolomics	SD	Kefale et al., 2023
RNA-seq	SD	Li et al., 2017	Metabolomics	SD	Li et al., 2022
RNA-seq	SD	Yang et al., 2020	QTL-seq and RNA-seq	Pod length	Lv et al., 2024
RNA-seq	Seed size	Wu et al., 2022	QTL-seq and RNA-seq	Seed weight	Wang et al., 2022
RNA-seq	Seed size	Li et al., 2021b	Metabolomics-Transcriptomics joint analysis	Seed coat color	Xue et al., 2021
RNA-seq	Seed size and Oil content	Guo et al., 2022	Metabolomics-Transcriptomics joint analysis	Seed coat color	Hu et al., 2021
RNA-seq	Oil Content	Wang et al., 2018a	Metabolomics-Transcriptomics joint analysis	Seed coat color	Wang et al., 2022
RNA-seq	Seed size and oil content	Yang et al., 2023	Metabolomics-Transcriptomics joint analysis	SD	Li et al., 2022
RNA-seq	Oleic acid content	Liu et al., 2018	Metabolomics-Transcriptomics joint analysis	Pod size	Lv et al., 2022
			Lipidomics and proteomicsjoint analysis	Oleic acid content	Liu et al., 2020

WGS-seq, Whole genome resequencing; BSA-seq, Bulked segregant analysis based on deep sequencing; SLAF-seq, Specific locus amplified fragment sequencing; ddRAD-seq, Double digest restriction-site associated sequencing; SD, Seed developmental.

## QTL mapping and GWAS analysis of peanut seed-related traits driven by re-sequencing data

The foundation for mapping QTLs associated with seed traits in peanuts has been laid through the use of a genetic map, where the quality and precision of QTL mapping, as well as the accurate localization of QTL regions, are significantly influenced by the number and density of markers. Recent research efforts employing specific locus amplified fragment sequencing (SLAF-seq) (Wang et al., 2018b; Zhang et al., 2019; Zhao et al., 2022), genotyping-by-sequencing (GBS) (Zhang et al., 2017), and double-digest restriction-site-associated DNA sequencing (ddRAD-seq) (Wang et al., 2019; Luo et al., 2021; Zhang et al., 2021) have contributed to the generation of over 2,000 SNP markers on genetic maps, underscoring the role of omics data in enhancing genetic analysis. With the declining cost of sequencing, whole-genome re-sequencing has emerged as a powerful approach for generating large-scale SNP markers and constructing high-density genetic maps. Notably, this approach has led to the development of four high-density genetic linkage maps, each containing over 8,000 SNPs (Agarwal et al., 2018, 2019; Liu et al., 2020b; Jiang et al., 2021), making a significant advance in the ability to identify markers on a large scale, especially in the context of low genetic diversity in peanut germplasm (Jiang et al., 2011; Wang et al., 2011; Mukri et al., 2012).

In recent genetic studies, various quantitative trait loci (QTL) associated with seed-related traits have been identified, offering insights into the complex genetic foundations of these traits. Noteworthy discoveries include the identification of one stable QTL linked to seed weight on the terminal regions of chromosome B07 (Wang et al., 2018b). Further research identified additional stable QTLs influencing seed weight located on chromosomes A02 and B06 (Zhang et al., 2019). In terms of nutritional traits, a major QTL, *qA05.1*, was found to have a significant impact on oil, protein, and six fatty acids across diverse environments, highlighting the intricate genetic interactions shaping the nutritional composition of peanuts (Sun et al., 2022). A detailed examination conducted by Hu et al. (2018) identified QTLs related to oleic acid (C18:1), linoleic acid (C18:2), and the oleic-to-linoleic acid ratio (O/L) on chromosomes A03, A04, A09, B09, and B10, illuminating the genetic regulation of fatty acid composition. Liu et al. (2020c) discovered a stable QTL, *qOCA08.1*, on chromosome A08, which explained a substantial proportion of phenotypic variation in oil content. Fine-mapping of this QTL revealed a ~0.8-Mb genomic region harboring two annotated genes influencing oil synthesis, providing vital insights into the genetic determinants of oil-related traits in peanut. Employing BSA-seq technology, Guo et al. (2023) uncovered four QTLs for sucrose content on chromosomes A03 and A06, while Wang et al. (2024) further identified two homologous QTLs on chromosomes A06 and B06, providing valuable information on the genetic factors impacting this essential trait. Furthermore, studies on color traits identified key genes controlling red testa color. QTL analysis and fine-mapping identified the *AhRt2* gene on chromosome 12, associated with a SNP in the third exon, as crucial for red testa color (Zhang et al., 2022b). Additionally, the *AhTc1* gene, encoding an R2R3-MYB transcription factor, was found to regulate purple testa color (Zhao et al., 2020b), while *AhRt1* was mapped to a region on chromosome A03, associated with a bHLH transcription factor gene, further elucidating the genetics underlying testa color in peanut (Chen et al., 2021).

Advances in genome-wide association study (GWAS) analyses have significantly contributed to the understanding of peanut seed traits. Zhao et al. (2022) identified SNP markers associated with hundred seed weight, branch number, and pod shape. In the Chinese peanut core collection, Zhou et al. (2021) uncovered two major loci exhibiting pleiotropic effects on yield-related traits, explaining about 20% of phenotypic variation. Furthermore, Zhou et al. (2022) identified three stable major associated loci, including two on chromosome A09 for oleic acid and linoleic acid and one on B06 for stearic acid. Extending the research to the USDA peanut core collection, Otyama et al. (2022) explored genetic markers tied to variations in fatty acid composition, unveiling 10 markers affecting oleic and linoleic acid contents, with the alleles having inverse impacts on these acid concentrations. Moreover, Zhang et al. (2017) uncovered 18 significant markers related to seed weight distributed across six chromosomes. Wang et al. (2019) further identified seven peak SNPs associated with yield per plant, pod weight, and seed weight. Zhang et al. (2021) identified five stable significant SNPs associated with oil content and three stable significant SNPs associated with C24:0. Collectively, these GWAS findings offer a comprehensive view of the genetic architecture underlying various peanut seed traits, facilitating targeted breeding efforts for improved cultivars.

## The regulation of peanut seed development based on transcriptome data

The study of peanut seed development through transcriptome analysis has led to significant insights, with numerous publications highlighting the intricate genetic networks involved. A pivotal study by Clevenger et al. (2016) sequenced a comprehensive transcriptome map covering 22 tissue types throughout the peanut’s reproductive development, from flowering to seed maturation. This work, in conjunction with additional RNA-seq data from Yin et al. (2013); Gupta et al. (2016), and Zhang et al. (2012), has provided a detailed gene expression landscape during seed development.

Peanuts exhibit a unique botanical feature: aerial flowering followed by subterranean fruit development. The failure of peg penetration into the soil inhibits the start of pod swelling, resulting in the development of aerial pods and ultimately leading to seed abortion. Comparative transcriptomic analyses between aerial and subterranean pods have identified genes associated with early embryo abortion, including up-regulated photosynthesis-related genes and senescence-associated genes in aerial pods, which may hinder pod swelling (Chen et al., 2013; Zhu et al., 2014). Further transcriptome analyses have identified crucial genes in the embryo and basal regions of the peg, both before and after soil penetration. These genes, including MADS-box transcription factors and cellulose synthase, are vital for embryo development and pod formation (Zhang et al., 2016). Chen et al. (2016a) expanded the research to encompass two whole pod stages and nine stages of isolated pod walls, revealing a developmental gradient of gene expression and highlighting the roles of transcription factors in pod development. Similarly, MADS-box transcription factors play a pivotal role in regulating seed development in both grapevine (Grimplet et al., 2016) and Arabidopsis (Simona et al., 2011), highlighting their fundamental importance across diverse plant species. Zhao et al. (2020a) explored alternative splicing in early swelling pods, finding it mainly related to ovule development, root hair cells enlargement, root apex division, and seed germination. Chen et al. (2019b) and Yu et al. (2015) focused on oil metabolism, identifying over 2,500 genes related to lipid biosynthesis. Their expression patterns during seed development offer insights into peanut lipid biosynthesis.

Studies on the dynamic transcriptomic changes during pod filling have shed light on genotypic variations in lipid metabolism and pod filling efficiency, with the “Hanoch” genotype showing superior pod-filling capabilities (Gupta et al., 2016). Liu et al. (2020a) examined the developmental transcriptome of underground peanut pods and identified 165,689 transcripts, revealing a shift from DNA synthesis and cell division to cell expansion and storage during seed development, with photosynthetic genes active in both aerial and subterranean pods. Moreover, the role of calcium, a crucial signaling molecule, in peanut pod development has been explored. Li et al. (2017) and Yang et al. (2020) investigated the effects of calcium deficiency on gene expression related to calcium signaling and hormone regulation during pod development. Similar to findings in peanuts, studies on wheat and Chinese cabbage reveal the pivotal role of calcium in sustaining plant health, underscoring how calcium deficiency influences gene expressions linked to calcium signaling and hormonal regulation in these diverse agricultural crops (Aslam et al., 2017; Zhang et al., 2022). These studies illustrate the complex interplay between calcium signal transduction, hormone pathways, and the genetic regulation of peanut seed development, providing valuable insights for targeted breeding and genetic improvement initiatives.

## The trait-related regulation of peanut seed development based on transcriptome data

Comprehensive transcriptomic analyses have shed light on the genetic mechanisms behind peanut seed development, size, and oil content. For instance, genes such as PNC, YUC, and GASA were found to influence auxin synthesis and seed size, while specific variant sites like GCP4 and RPPL1 within QTL intervals play roles in cell tissue microtubule nucleation (Wu et al., 2022). These findings were consistent with the research in other plants (Cao et al., 2020), which highlighted the importance of auxin and related genes in seed development and grain yield. RNA-seq data from cultivated peanuts and wild Arachis monticola identified genes uniquely expressed during seed development, with certain proteins potentially linked to increased seed size in cultivated varieties (Li et al., 2021b). Differences in gene expression between genotypes with varying seed size and oil content have identified pathways related to plant hormones and fatty acid biosynthesis as critical for seed related traits (Guo et al., 2022).

In terms of oil content, the analysis of 49 cultivars uncovered significant markers on chromosome A03, aiding marker-assisted selection in breeding (Wang et al., 2018a). Comparative studies between peanut varieties with different seed sizes and oil levels identified genes and networks involved in fatty acid synthesis, suggesting strategies for improving seed yield and quality (Yang et al., 2023). Similar to research conducted on peanuts, studies in other crops like safflower, soybean, and rapeseed have also explored key genes and networks involved in regulating traits such as like seed size, oil content, and fatty acid composition (Fan et al., 2023; Guan et al., 2023; Zhao et al., 2023). Research on oleic acid content between cultivars highlighted the role of FAB2 in unsaturated fatty acid biosynthesis and lipid oxidation (Liu et al., 2018), which agreed with the reports in other plants (Dar et al., 2017; He et al., 2020).

For sucrose content, comparative transcriptomics between high- and low-sucrose peanut varieties revealed genes linked to sucrose metabolism, offering targets for molecular breeding (Li et al., 2021a). Studies on seed coat color have utilized transcriptomics to identify genes and markers associated with testa color, providing valuable information for breeding peanuts with desired coat characteristics (Wan et al., 2016; Huang et al., 2020). These findings collectively enhance our understanding of peanut genomics and support targeted breeding efforts for trait improvement.

## Deciphering peanut seed development based on proteomic, metabolic, and epigenetic data

The exploration of peanut seed development through integrated approaches combining proteomics, metabolomics, and epigenetics has yielded profound insights into the molecular underpinnings of key seed traits such as size, oil content, allergenicity, and amino acid composition.

Proteomic analyses have significantly advanced our understanding of the dynamic protein changes during peanut seed development. Studies have identified a diverse array of proteins involved in carbohydrate, amino acid, and lipid metabolism, highlighting their crucial roles in seed development (Wang et al., 2016; Li et al., 2020). Some of the proteins identified during the development of peanut seeds were also found in other crops such as rice (Yoon et al., 2023), soybeans (Wei et al., 2020), wheat (Dong et al., 2015), and barley (Chen et al., 2022). Notably, the identification of allergen proteins and their expression patterns offers valuable insights into allergen accumulation processes, informing breeding strategies aimed at reducing allergenicity (Li et al., 2020). Moreover, the differential expression of proteins related to lipid metabolism during seed development and post-germination stages underscores the complex regulatory mechanisms governing oil accumulation and degradation (Wang et al., 2016).

Epigenetic modifications, specifically DNA methylation, have been elucidated as pivotal regulatory mechanisms influencing peanut seed development. Comparative analyses have demonstrated global methylation changes accompanying seed development, with significant correlations between methylation levels and gene expression, particularly in pathways related to seed size and oil content (Liu et al., 2022; Li et al., 2023). These findings suggest that epigenetic regulation plays a substantial role in modulating seed trait expression.

The regulation of gene expression during seed development has also been a focus, with studies revealing the importance of circRNAs and miRNAs in this process. CircRNAs have been implicated in seed development and size regulation, pointing to their involvement in post-transcriptional regulatory networks (Feng et al., 2019). Similarly, miRNA-mediated regulatory networks have been identified as key contributors to embryo development under calcium deficiency and seed expansion, highlighting the roles of specific miRNAs in modulating gene expression related to growth and development processes (Ma et al., 2018; Chen et al., 2019a).

Metabolomics has recently been embraced in peanut research, offering novel insights into our understanding of its seed develpment. Zhang et al. (2022) employs UPLC-MS/MS to profile metabolites in the testa of four peanut germplasms with varied colors, identifying 85 metabolites and highlighting the significant diversity and differential accumulation of these compounds, including proanthocyanidins, isoflavones, flavonols, and anthocyanidins (Zhang et al., 2022a). Kefale et al. (2023) identified many differentially accumulated metabolites related to amino acid metabolism, phenylpropanoid biosynthesis, flavonoid biosynthesis, and lipid metabolism between peanut and other oil crops. Li et al. (2022) found that during the early stages of development, most amino acids were present at significantly lower levels. However, this trend shifted in the middle and late stages, where the levels of amino acids were notably higher.

## Deciphering peanut seed development and seed-related traits based on joint multi-omics analysis

The joint multi-omics analysis, including genomics-transcriptomics, metabolomics-transcriptomics, proteomics-transcriptomics, serves as a powerful toolkit for elucidating the mechanisms regulating peanut seed development and controlling seed-related traits. In terms of genomics-transcriptomics joint analysis, QTL-seq and RNA-seq have been successfully applied to identify candidate genes for pod length (Lv et al., 2024) and seed weight (Wang et al., 2022). For metabolomics-transcriptomics joint analysis, three studies untilize a integrated approach to investigate the regulatory mechanisms behind testa pigmentation in peanuts. Xue et al. (2021) led this exploration, uncovering the intricate anthocyanin metabolism, highlighting the importance of petunidin 3-O-glucoside and cyanidin O-acetylhexoside in color differentiation. Their analysis identified crucial genes and transcription factors, such as CHS, DFR, MYB, bHLH, and WD40, as pivotal in regulating the distinct pigmentation of peanut testa. Hu et al. (2021) further analyzed the flavonoid biosynthesis pathway, identifying 27 significantly differentially expressed genes (DEGs) associated with testa color development, emphasizing the roles of cyanidin and delphinidin. Wang et al. (2022) broadened this investigation by profiling 133 flavonoids across four peanut cultivars, correlating specific flavonoid components with a variety of testa colors and detailing the roles of cyanidin-based anthocyanins, MYB-like transcription factors, anthocyanidin reductases (ANR), and UDP-glycosyltransferases (UGT) in color modulation (Wang et al., 2022). Additionally, transcriptomic and metabolomic analyses have shed light on the genetic and metabolic pathways involved in seed development, with the identification of genes and proteins involved in amino acid metabolism, notably arginine biosynthesis, providing avenues for enhancing the nutritional quality of peanut seeds (Li et al., 2022). Lv et al. (2022) identifies that the accumulation of p-coumaryl alcohol and its associated biosynthesis pathway, particularly the differential expression of gene LOC112771695, plays a critical role in determining peanut pod size. By integrating lipidomics and proteomics, Liu et al. (2020) unravel the complex dynamics of lipid molecular species and their association with the FAD2 mutation in high-oleic acid peanut seeds.

## Perspectives

This review demonstrates the pivotal role of omics technologies and related data in achieving a comprehensive peanut reference genome and deepening our insight into the regulatory mechanisms governing peanut seed development. Extensive analysis across various developmental phases, encompassing gene expression, proteomics, metabolomics, and epigenetics, has unveiled the molecular underpinnings and identified key regulatory mechanisms and QTLs linked to seed traits. Such discoveries have substantially contributed greatly to our comprehension of peanut seed development and trait regulation. However, there is still a relative scarcity of research on the cloning and functional study of peanut functional genes. Accelerating the fine mapping of QTLs and employing multi-omics techniques to identify functional genes are essential next steps. Additionally, the application of gene-editing technologies for the improvement of seed traits and the creation of new germplasms represents a crucial research direction for the future. The genome navigation system, including platforms like RiceNavi (Wei et al., 2021), was anticipated to be developed for the purpose of QTN pyramiding and optimizing breeding routes in peanuts. These approaches will not only deepen our understanding of peanut biology but also facilitate the breeding of varieties with enhanced yield, quality, and environmental resilience.

## Author contributions

ZW: Writing – original draft, Writing – review & editing. YL: Writing – original draft, Writing – review & editing. BL: Writing – original draft, Writing – review & editing.
